# The association between Roussouly sagittal alignment type and risk for adjacent segment degeneration following short-segment lumbar interbody fusion: a retrospective cohort study

**DOI:** 10.1186/s12891-022-05617-x

**Published:** 2022-07-08

**Authors:** Zhe Qu, Bin Deng, Xiao Gao, Bin Pan, Wei Sun, Hu Feng

**Affiliations:** 1 Department of Spine Surgery, the Affiliated Hospital of Xuzhou Medical University, Huaihai West Road 99, Xuzhou, 221006 China; 2 Xuzhou Medical University, Xuzhou, China

**Keywords:** Adjacent segment degeneration, Radiological ASD, Roussouly type, Spinopelvic parameters, Lumbar interbody fusion

## Abstract

**Background:**

To date, the influence of Roussouly type on development of adjacent segment degeneration (ASD) after lumber fusion is still not fully explored, and the current study is aimed to evaluate the effect of Roussouly type on development of radiological ASD after single-level lumber fusion, and to compare the Roussouly types and spinopelvic parameters among those with different degenerative patterns of ASDs on sagittal plane.

**Methods:**

A retrospective review of 288 patients underwent L4/5 or L5/S1 single-level posterior interbody fusions between January 2016 and December 2018 with a minimum 2-year follow up was performed. Radiological ASDs were identified and divided into 3 groups according to different degenerative patterns of the cephalad adjacent level on sagittal plane, including the types of retrolisthesis (Group A), anterolisthesis (Group B), and axial disc space narrowing (Group C). Roussouly types and radiological measurements were compared among three groups and potential risk factors for ASD were evaluated.

**Results:**

Radiological ASD was found in 59 (20.5%) cases, in which patients with Roussouly type-2 was the most common. While, on subgroup analysis among three ASD groups, Roussouly type-1 occupied the highest proportion in Group A, differ in Group B and Group C, both with Type-2 as the most common. Moreover, Group A had significantly lower pelvic tilt (PT), larger sacral slope (SS), and larger segmental angle (SA) than Group B and Group C, which showed a more anteverted pelvic in Group A. Multivariate regression analysis noted Roussouly type, preoperative PT, and ∆PI-LL as the independent risk factors for radiological ASD.

**Conclusion:**

Roussouly type was significantly associated with the development of radiological ASD; however, the Roussouly types and spinal pelvic parameters were varied among different sagittal degenerative patterns of ASD, which was important in restoring optimal lumbar sagittal alignments in initial surgery.

## Background

Posterior lumbar arthrodesis surgery, mainly comprised of posterior lumber interbody fusion (PLIF) and transforaminal lumber interbody fusion (TLIF), has acquired favorable clinical outcomes; however, the solid fusion of naturally mobile vertebral segments may distort the stress distributions on adjacent non-fused segments and alter the kinematic patterns of these levels, leading to adjacent segment degeneration (ASD) [1, 2]. Even as one of the most important complications that affect long-term outcomes after PLIF/TLIF, the majority of ASDs are asymptomatic and detected on routine radiological examinations. The reported incidence of radiographic ASD varied from 8.7 to 72.7%, which was 2 to 10 times more than symptomatic ASD [3].

The development of ASD is multifactorial, previous studies noted that older age, obesity, preoperative degeneration of adjacent segments, more fusion levels, excessive distraction of the disc space, lamina horizontalization, facet tropism, and failure to restore lumbar lordosis may increase the risk of ASD [4–6].. In last decades, spinopelvic parameters and sagittal spinal profiles were also found to be important predictors of ASD [7–10]. To describe the sagittal spinal profile, Roussouly classified the lumbar spine into four different types based on sacral slope and sagittal shapes [11]. Even though Roussouly type was originally determined in healthy adults, it was also helpful in the evaluation of lumbar degenerative diseases [12]. Recently, Duan et al. confirmed the revision rate was highest in Roussouly Type-2 patients for symptomatic ASD after L4/5 interbody fusion with 2-year follow-up [13]; however, the effects of Roussouly classification on the development of radiographic ASD remains uncertain.

As most ASDs are identified on radiographs during follow-up, there are three main degenerative patterns of ASDs on sagittal plane that can be identified, including retrolisthesis or anterolisthesis of the adjacent segment, and axial disc space narrowing [14–16]. While, the radiological and biomechanical features might be different among these three types of degenerative patterns, and the influences of Roussouly types and spinopelvic parameters on development of ASDs with different sagittal degenerative patterns are still unexplored.

Therefore, the current case-control study is aimed to associate Roussouly sagittal profile types with radiological ASDs in patients underwent single-level PLIF/TLIF; and to compare the sagittal spinal profiles and spinopelvic parameters among ASD patients with three degenerative patterns on sagittal plane.

## Methods

### Subjects

After institutional review board approval, a retrospective review was conducted to identify all patients who underwent single-level lumbar interbody fusion surgeries for degenerative diseases at our hospital from January 2016 to December 2018. Inclusion criteria for the study were: (1) patients diagnosed with L4/5 or L5/S1 single-level degenerative spinal diseases, including spinal stenosis, disc herniation, and grade I spondylolisthesis; (2) TLIF or PLIF surgery was performed at L4/5 or L5/S1; (3) availability of preoperative radiological examinations including plain films in standing position and lumbar MRI, as well as standing lumbar radiographs at the final follow-up; and (4) a minimum follow-up duration of 2 years. Patients were excluded due to: (1) fusion of more than one level, or at the levels cephalad to L4/5; (2) preexisting severe degeneration or spondylolisthesis at adjacent segments; (3) anterior or lateral lumbar fusion; (4) lumbar scoliosis more than 10°; (5) grade II or more severe degenerative spondylolisthesis; (6) patients with spondylolysis, spinal infection, vertebral fracture, or spinal tumor. Demographic data of the patients enrolled were collected.

During the follow-up period, standing radiographs of the lumbar spine were routinely obtained. Radiographic documentations at the last visits of those with more than 2 years follow-up or those presented symptomatic ASD were investigated. Radiographic ASD was defined as development of retro- or anterolisthesis ≥3 mm of the cephalad adjacent vertebrae or reduction of ≥50% in cephalad adjacent disc height on the neutral lateral radiograph in the free-standing position [17, 18]. In the present study, we limited the investigation on cranial adjacent segment for two reasons: one lies in the fact that the cephalad level is at higher risk for ASD, accounting for more than 80% cases [3]; another was that only L4/5 or L5/S1 fusions were included in our study, a high proportion of L5/S1 fusions further limited the possibilities of developing ASDs at the caudal level.

### Data collection

**Fig. 1:**
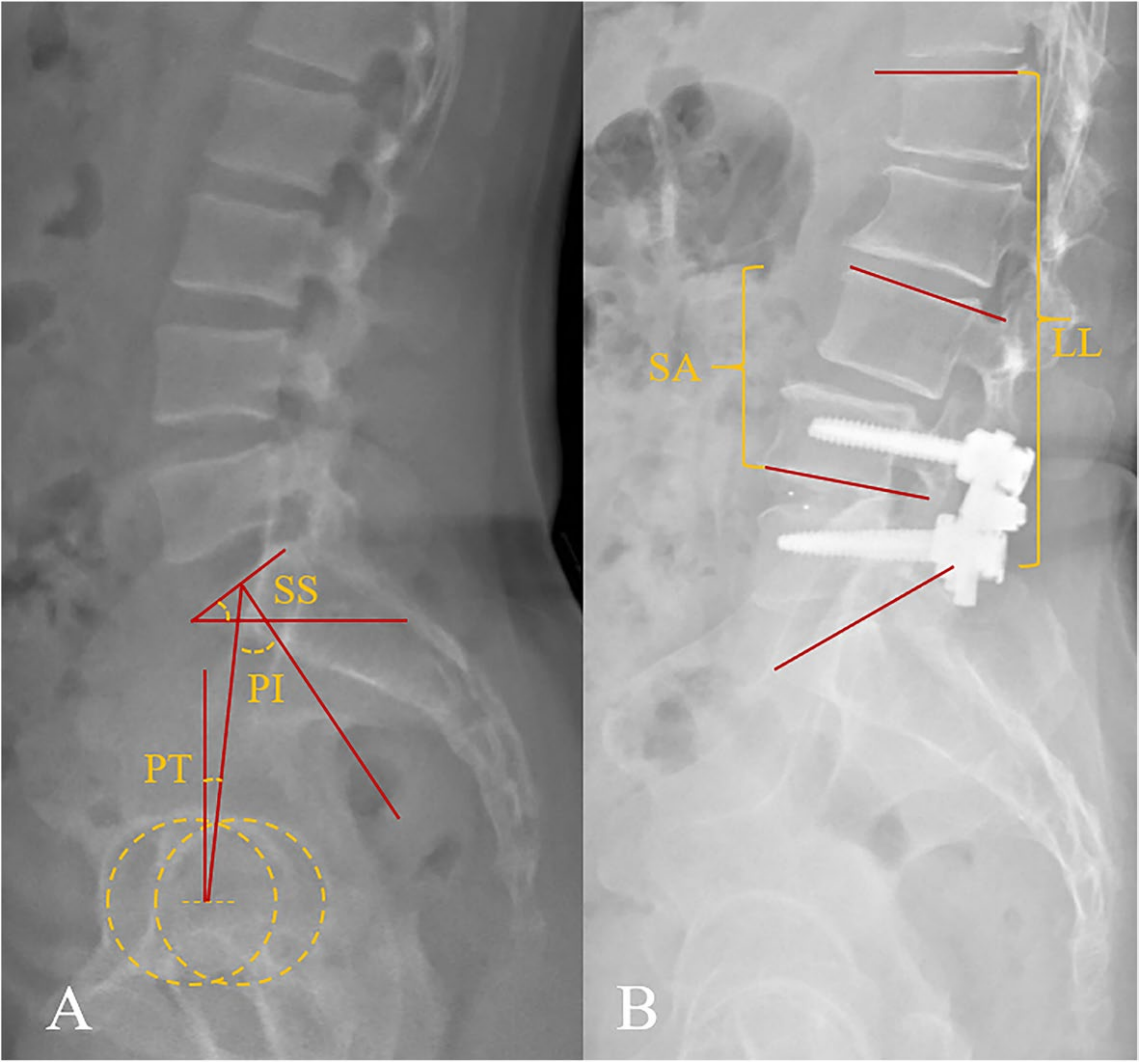
Measurement of radiographic parameters. **A.** spinopelvic parameters: Pelvic Incidence (PI) is measured as the angle between the line perpendicular to the middle of sacral plate and the line connecting the hip axis and midpoint of sacral plate; Pelvic Tilt (PT) is the angle between the vertical line and the line joining hip axis and the middle of sacral plate; Sacral Slope (SS) is formed by the endplate of S1 and the horizontal line. **B.** Lumbar Lordosis (LL) is regarded as the angle between the upper endplates of L1 and S1, using the Cobb method; Segmental Angle (SA) is defined by the Cobb angle between the upper endplate of the cephalad adjacent vertebra and the lower endplate of the upper instrumented vertebra

Radiological assessments were conducted utilizing standard free-standing lateral radiographs preoperatively and at final follow-up. Measurements consisted of spinopelvic parameters, such as pelvic incidence (PI), pelvic tilt (PT), sacral slope (SS), lumbar lordosis (LL), and segmental angle (SA) of the cephalad adjacent level. In the current study, LL was defined as the Cobb angle between the superior endplates of L1 and S1; and SA was measured as the angle between the lower endplate of the proximal fused vertebra and the upper endplate of the cephalad adjacent vertebra; moreover, ∆PI-LL was calculated by the difference between PI and LL [19]. Measurements of these parameters were depicted in Fig. 1. All parameters were measured for three times by independent orthopedic residents, the average of the three measurements were calculated. To estimate the reliabilities of these measurements, intraclass correlation coefficient (ICC) was applied. The overall ICC for the measures was 0.87, indicating satisfactory agreements.

Sagittal alignment of the lumbar spine was categorized into 4 types using Roussouly method on preoperative radiographs [11]: Type-1 patients had a long thoracolumbar kyphosis and a short lumbar lordosis with SS ≤ 35°; Type-2 also with low SS, but the sagittal profile showed a fat back appearance; SS in Type-3 was between 35° and 45°, with an almost equal length of kyphosis and lumbar lordosis curves in these patients; Type-4 with the SS ≥ 45°, showed a long lordosis and consecutive shorter kyphosis. On preoperative MRI, disc degeneration of the adjacent level cephalad to the fused segment was assessed according to Pfirrmann’s grading system, which was classified into 5 grades based on intervertebral disc structure, distinction of nucleus and anulus, signal intensity, and the height of intervertebral disc on T2-wighted midsagittal images [20].

In ASD patients, three groups of degenerative patterns on lateral lumbar films at the last follow-up were divided: posterior spondylolisthesis of the cephalad adjacent vertebra (Group A), anterior spondylolisthesis (Group B), and disc space narrowing without significant spondylolisthesis (Group C); and the disparities in Roussouly types and spinopelvic parameters among three groups were compared. In addition, risk factors associated in the development of ASD were analyzed include the following confounding factors: Roussouly sagittal profile type, preoperative Pfirrmann grade at the adjacent level, spinopelvic parameters, and other related variables. For the categorical data, including Roussouly type, ASD classification, and Pfirrmann grade, classifications were first performed by two independent senior spine surgeons (DB and SW), if they got the inconsistent results, the third senior surgeon (FH) was invited to confirm the classifications with other two surgeons, by which the reliabilities were assured.

### Statistical analysis

Statistical analysis was conducted using SPSS version 19.0 (IBM, Chicago, IL). Categorical variables were compared utilizing either chi-square or Fisher’s exact tests, and the continuous data among different groups were compared via student’s t-test or one-way ANOVA test. Univariate and multivariate logistic regression analyses were performed to identify the independent risk factors for ASD, variables with p<0.1 in univariate analyses were considered as potential risk factors which were taken into the final multivariate logistic regression model. In the current study, the two-tailed significance level was set at p<0.05.

## Results

### Enrolled subjects

Between January 2016 and December 2018, 518 patients underwent L4/5 or L5/S1 single-level interbody fusion for degenerative lumbar diseases were primarily enrolled in our study. Among them, 162 patients were excluded for less than 2 years follow-up. From the remaining 356 patients, radiological data were unavailable or incomplete in 68 cases, and hence, a total of 288 patients fit the inclusion and exclusion criteria were included finally.

Of the 288 patients included, 112 were males and 176 were females, with a mean age of 57.9 years (range, 41–82 years). These patients were followed up from 24 months to 61 months (30.2 months on average). During the follow-up period, 59 out of the 288 patients developed radiological ASD with an incidence of 20.5%. These 59 patients formed the ASD group, and the rest 229 cases formed the control group. All surgeries were performed by three senior spine surgeons (DB, SW, and FH), including 37 TLIFs and 22 PLIFs in ASD group, and 173 TLIFs and 56 PLIFs in the control group. The surgeries were following standard TLIF/PLIF procedures, techniques of MIS-, ENDO-, or unilateral- procedures were not applied in the current study.

The post-operative complications of those included were found to be the following: superficial surgical site infection in 9 cases, deep infection in 6 cases, wound disruption in 4 cases, neurological compromise in 5 cases, acute cerebral infarction in 2 cases, and urinary tract infection in 3 cases. Superficial wound infection and the 2 deep infection patients received regular anti-infective therapy, other 4 deep infectious patients underwent additional surgical debridement and drainage. Four wound disruption cases received wound debridement and suturing to promote healing. Reoperations were performed in the 5 patients with neurological deficits, surgical exploration identified hematoma in 3 and misplaced pedical screws in 2, hematoma clearance or revision of the instrumentations were then carried out. Cerebral infarction and urinary tract infection patients were treated with thrombolytic therapies and anti-infective therapies. Satisfactory outcomes were obtained after aforementioned treatments of all patients with post-operative complications, leaving no sequela.

### Comparisons of the baseline data among ASD cases and controls

The detailed patient demographics and pre-operative spinopelvic data are shown in Table 1. No significant difference was observed in age, sex, height, weight, and BMI between ASD and control group. Segments operated in ASD group including L4/5 in 34 cases and L5/S1 in 25 cases, the control group had 135 L4/5 fusions and 94 L5/S1 fusions. For Pfirrmann’s grade of the cephalad adjacent level, Grade I, Grade II, Grade III, and Grade IV in ASD group were 15, 23, 12, and 9 cases, respectively; in control group, Pfirrmann’s Grade I, Grade II, Grade III, and Grade IV were 63, 62, 56, and 48 cases, respectively. Both the fusion levels (*P =* 0.88) and Pfirrmann’s grade (*P =* 0.43) were comparable between the two groups.

**Table 1:** Demographic and pre-operative spinopelvic data of all patients

	ASD group	Control group	*P*-value
Males/Females (n)	25/34	87/142	0.55
Age (yrs.)	58.2 ± 8.1	57.9 ± 8.4	0.75
Height (cm)	171.4 ± 8.5	166.7 ± 9.2	0.15
Weight (kg)	72.3 ± 9.5	68.5 ± 11.3	0.23
BMI (kg/m^2^)	24.6 ± 2.4	24.4 ± 2.7	0.42
Fusion level (n)			0.88
L4/5	34	135	
L5/S1	25	94	
Pfirrmann’s grade (n)			0.43
Grade I	15	63	
Grade II	23	62	
Grade III	12	56	
Grade IV	9	48	
Roussouly type (n)			0.032^a^
Type-1	17	36	
Type-2	21	65	
Type-3	9	60	
Type-4	12	68	
PI (°)	48.9 ± 9.0	49.4 ± 8.3	0.72
PT (°)	20.6 ± 4.9	17.0 ± 5.0	0.029^a^
SS (°)	32.4 ± 8.0	35.4 ± 9.1	0.021^a^
LL (°)	34.2 ± 7.8	39.7 ± 12.3	0.01^a^
SA (°)	9.8 ± 4.6	9.0 ± 4.3	0.23

^a^ Indicates statistically significant difference

When refer to the sagittal profile, the most common Roussouly type in ASD group was Type-2 (35.6%), followed by Type-1 (28.8%), Type-4 (20.3%), and Type-3 (15.3%); while, the distributions were significantly different in control group (*p =* 0.032), with the most common type of Type-4 (29.7%), followed by Type-2 (28.4%), Type-3 (26.2%) and Type-1 (15.7%). In terms of spinopelvic parameters, no significant difference was found in PI (48.9° vs 49.4°, *p =* 072), but ASD group showed significantly higher PT (20.6° vs 17.0°, *p =* 0.029), lower SS (32.4° vs 35.4°, *p =* 0.021), and LL (34.2° vs 39.7°, *p =* 0.01).

In comparisons of the parameters before and after the development of ASD in ASD group, no significant difference of PT, SS, and LL was observed; while, only SA in the last follow-up was significantly larger than that preoperatively (9.8° vs. 11.1°, *P =* 0.03).

### Roussouly type distribution and spinopelvic parameters in ASD patients with different degenerative patterns

In ASD group, the most common degenerative pattern of the adjacent level on sagittal plane was the retrolisthesis type (Group A, *n =* 25), followed by the disc space narrowing (Group C, *n =* 18) and anterolisthesis type (Group B, *n =* 16). The distributions of Roussouly types in different groups are shown in Table 2, and significant differences of the distributions among three groups were noted (*p =* 0.033). In Group A, Roussouly Type-1 was most frequently seen (48%), and Roussouly Type-4 (28%) was at the second place; however, in both Group B and Group C, Roussouly Type-2 occupied the highest proportions, accounting for 50% in each group.

**Table 2:** Distribution of Roussouly types by different degenerative patterns of ASD patients

	Roussouly type				*P*-value
	Type-1	Type-2	Type-3	Type-4	
Group A (*n =* 25)	12	4	2	7	0.033^a^
Group B (*n =* 16)	3	8	3	2	
Group C (*n =* 18)	2	9	4	3	

^a^ Indicates statistically significant difference

Sagittal spinal and spinopelvic parameters were compared among the three groups pre- and post-operatively (Table 3). No significant difference of PI was observed (*p =* 0.51). Preoperative average PT was 11.2° in Group A, which was significantly lower than that in Group B (15.8°) and Group C (15.0°, *p =* 0.003); correspondingly, preoperative SS in Group A (36.2°) was higher compared with Group B (29.1°) and Group C (30.1°, *p =* 0.006). The similar trends were shown at postoperative measurements, as the Group A had significantly lower PT (10.3° vs 14.6° vs 14.0°, *p =* 0.001) and higher SS (37.1° vs 30.3° vs 31.6°, *p =* 0.007) at the final follow-up. No obvious difference was found in LL and ∆PI-LL among three groups both pre- and post-operatively. As for SA, there was a significantly higher preoperative value in Group A (11.6° vs 7.9° vs 8.9°, *p =* 0.023), similarly, the postoperative SA in Group A was higher compared with Group B and Group C (13.5° vs 9.1° vs 9.4°, *p =* 0.01).

**Table 3:** Comparison of radiological measurements among different groups of ASD degenerative patterns pre- and post-operatively

	Pre-op						Post-op				
	PI	PT	SS	LL	∆PI-LL	SA	PT	SS	LL	∆PI-LL	SA
Group A	47.3	11.2	36.2	34.1	13.2	11.6	10.3	37.1	37.2	10.3	13.5
Group B	49.8	15.8	29.1	33.5	16.3	7.9	14.6	30.3	36.4	13.5	9.1
Group C	50.3	15.0	30.1	34.9	15.5	8.9	14.0	31.6	37.4	13.0	9.4
*P*-value	0.51	0.003^a^	0.006^a^	0.89	0.10	0.023^a^	0.001^a^	0.007^a^	0.93	0.06	0.01^a^

^a^ Indicates statistically significant difference

### Risk factor analysis for ASD

Variables included demographic data, height, weight, BMI, Pfirrmann’s grade, Roussouly type, and spinopelvic parameters were plug into logistic regression analysis. Univariate analyses demonstrated that the following factors were significantly associated with the development of ASD: Roussouly type (*p =* 0.034), preoperative PT (*p =* 0.02), preoperative ∆PI-LL (*p =* 0.023), postoperative PT (*p =* 0.003), postoperative SS (*p =* 0.04), postoperative ∆PI-LL (*p =* 0.04), and postoperative SA (*p =* 0.03). Even without statistical significance, preoperative SS showed trends of correlation with *p <* 0.1. The results of univariate analysis are shown in Table 4.

**Table 4:** The results of univariate analyses

Variables	Odds Ratio (95% CI)	*P*-value
Age	1.01 (0.97–1.04)	0.75
Sex	1.20 (0.67–2.15)	0.54
BMI	1.031 (0.976 ~ 1.090)	0.28
Fusion level	1.06 (0.59–1.89)	0.85
Pfirrmann’s grade	0.89 (0.68–1.16)	0.39
Roussouly type	Ref.	0.034**
Roussouly type (a)	2.68 (1.15–6.21)	0.022**
Roussouly type (b)	1.83 (0.83–4.02)	0.09*
Roussouly type (c)	0.85 (0.34–2.16)	0.73
PI	0.99 (0.96–1.03)	0.72
PT _(pre-op)_	1.52 (1.03–1.98)	0.02**
SS _(pre-op)_	0.96 (0.73–1.10)	0.08*
LL _(pre-op)_	0.98 (0.93–1.04)	0.62
∆PI-LL _(pre-op)_	1.08 (1.01–1.15)	0.023**
SA _(pre-op)_	1.04 (0.98–1.11)	0.23
PT _(post-op)_	1.09 (1.03–1.17)	0.003**
SS _(post-op)_	0.82 (0.79–0.96)	0.04**
LL _(post-op)_	0.94 (0.81–1.27)	0.73
∆PI-LL _(post-op)_	1.09 (1.03–1.16)	0.04**
SA _(post-op)_	1.10 (1.04–1.18)	0.03*

* *p<*0.1, ** *p<*0.05

Multivariate logistical regression analysis was performed to detect the independent risk factors for radiological ASD. Collectively, univariate factors with *p <* 0.1 were potentially associated with ASD and were included in the multivariate analysis. Moreover, to dispel the effects of multicollinearity between pre- and post-operative measurements, only preoperative PT, SS, and ∆PI-LL were included in the final regression model. As shown in Table 5, the Roussouly type (*p =* 0.039), preoperative PT (*p =* 0.041) and ∆PI-LL (*p =* 0.021) were identified as independent risk factors for ASD.

**Table 5:** The results of multivariate analyses

Variables	Odds Ratio (95% CI)	*P*-value
Roussouly type	Ref.	0.039^a^
Roussouly type (a)	2.55 (1.52–4.60)	0.03^a^
Roussouly type (b)	1.13 (0.40–2.23)	0.08
Roussouly type (c)	1.55 (0.52–4.59)	0.43
PT _(pre-op)_	1.28 (1.01–1.52)	0.041^a^
∆PI-LL _(pre-op)_	1.93 (1.75–2.28)	0.021^a^

^a^ Indicates statistically significant difference

## Discussion

The reported incidence of ASD following single level PLIF/TLIF at 2 years follow-up was varied from 11.7 to 22% [3], which was comparable with our present study with an incidence of 20.5%. The formation of ASD is multifactorial, many authors believed that the loss of naturally mobile segments after lumbar fusion may distort the distribution of forces on adjacent non-fussed levels and accelerate the degenerative change [21, 22]. In addition to mechanical loading, altered kinematics of the adjacent segments has been suggested as another crucial factor [17]. Except for aforementioned factors, spinopelvic parameters are essential for the mobile spine to adapt to the pelvis in order to achieve a mechanically efficient posture, which would also be involved in the process of ASD. In comparison of the spinopelvic parameters between ASD and control group in our study, significantly larger pre-operative PT, smaller SS, and smaller LL were manifested in the ASD cohort. Further multivariate logistical regression analysis also confirmed larger pre-operative PT and ∆PI-LL were independent risk factors for radiological ASD.

Of all the spinopelvic parameters, PI is constant and of primary importance in regulating lumbar sagittal alignment and predicting appropriate amount of LL [23]. Some authors thought that the loading patterns of the lumbar spine could be different based on PI, which was relevant for the development of ASD [24]. In our study, although PI was not found to be an independent predictor, the increasing ∆PI-LL was predisposed to ASD. From one biomechanical study, Senteler et al. reported that higher PI-LL mismatch exhibited higher shear forces on the adjacent level, with ∆PI-LL more than 15° had a 20 times higher risk for ASD [25]. Patients included in this study undertook different levels of fusions, more recently, Kim et al. evaluated the development of ASD after 4 levels lumbar fusions, they found PI-LL mismatch had significant correlations with radiological ASD, and restoration of optimal sagittal alignment could reduce ASD [26]. Similarly, we confirmed ∆PI-LL was also significantly associated with radiological ASD after single-level fusion. Therefore, when treating patients with high PI, hypolordosis of the instrumented segments may increase the loading forces of the adjacent level, and attention should be paid to achieve the appropriate LL.

PT represents a main compensatory mechanism of the pelvis in response to lumbar degeneration, and a large PT is associated with decreased spinal functions [27]. Matsumoto et al. evaluated the effects of spinopelvic balance on symptomatic ASD after single-level PLIF, they found higher preoperative PT was significantly associated with ASD [28]. Except for PT, SS was thought to have similar effects. In one retrospective study with 15 years follow-up, Maruenda et al. reported that patients with PT above 21° and SS below 39° were at higher risks for symptomatic ASD [29]. Phan et al. conducted a meta-analysis, and a significantly larger preoperative PT and smaller SS in the ASD group were determined [30]. Our univariate analysis noted both pre-operative PT and SS were associated with ASD; however, only PT was found to be the independent risk factor by multivariate regression analysis. A larger PT indicate more retroverted pelvic, which is related to suboptimal outcomes and sagittal imbalance after surgery [31]. We can infer from our results that patients with more posterior and horizontal inclinations of the pelvic, featured by larger PT and smaller SS, would bear more stress on intervertebral discs, accelerating the degenerations of the adjacent unfused segments, which contributed to the development of ASD.

In 2005, Roussouly et al. classified the sagittal spinal profile into four types depended on the shape of lumbar lordosis and the angulation of the pelvis [11]. Researchers believed that patients with different sagittal profiles have different biomechanical features that predispose patients to certain pathological changes [24]. Recently, Duan et al. firstly investigated the correlations between Roussouly type and revisions for ASDs after single-level TLIF, found that the Type-2 spine with PI-LL mismatch had the highest rate of revision surgery [13]. In the current study, we compared the distributions of Roussouly types between ASD group and control group, and identified the Roussouly Type-2 as the most commonly seen in radiological ASD group; Moreover, further regression analysis confirmed that Type-2 spine was the independent risk factor of radiological ASD. While, the effects of Roussouly types on the development of different types of ASDs could be different, as there were three main ASD types at the immediate cephalad segments on standing lateral radiographs obtained from routine follow-up, including types of anterior translation, posterior translation, and disc space narrowing. According to Aota et al. [14] and Kumar et al. [16], the most frequent type was the retrolisthesis of the adjacent vertebra, accounting for 60 and 48.4% of ASD patients, respectively. Similarly, we reported retrolisthesis type (42.4%) as the most common, followed by disc space narrowing (30.5%) and anterolisthesis (27.1%). Our study was the first to investigate the influences of Roussouly types and spinopelvic parameters on the development of different types of ASDs.

Significant differences of the distributions of Roussouly Types among three ASD groups were found. In both the anterolisthesis and disc space narrowing groups, Roussouly Type-2 was most commonly seen; however, in those with retrolisthesis of the cephalad vertebra, Roussouly Type-1 made up the largest proportion, followed by Type-4. The low PI in Type-2 spine indicated a limited ability to compensate for the sagittal alignment changes after lumbar fusion, and the compensations are mainly occurred at the adjacent unfused levels instead of the pelvic retroversion in those with a large PT; Moreover, as the Type-2 curve is a flat back with high loading forces at the adjacent levels, which accelerate the degenerative process of the adjacent level, inducing especially the disc space narrowing or anterolisthesis types of ASDs. In Type-1 curve, the apex of lumbar lordosis often located at the L5 vertebral body, when L4/5 or L5/S1 is instrumented and fused, the cephalad adjacent level situated in the junctional area between long thoracolumbar kyphosis and short distal lordosis, leaving the adjacent vertebra bear high shearing stress. While, for Type-4 lordosis patients, the underlying mechanisms in the development of the backward slipped ASDs might be different. Type-4 spine is characterized by a hypercurved lumbar with a high PI, when the optimal LL is not restored in short-segmental fusion, over extensions at the adjacent flexible levels would be occur to compensate for the local segmental hypolordosis, which increase the posterior sliding forces at the adjacent vertebra and develop into ASD finally. The larger SA in retrolisthesis group indicated a more tilted endplate of the upper instrumented vertebra, inducing a high risk of retrolisthesis. Therefore, during the surgical treatment of Roussouly Type-1 patients, it is important to avoid the segmental hyperlordosis at the fused level and to keep the upper instrumented vertebra at a horizontal sagittal place. Differently, restoring an optimal PI-LL should be of the greatest importance in the procedure of Type-4 spine, getting a large segmental lordosis at the fused level is necessary to avoid the over extension of the adjacent flexible level. In these cases, anterior or lateral interbody fusions using a large cage with a particular angle would be beneficial in restoring LL.

In comparisons of the spinopelvic parameters among three ASD groups, the retrolisthesis group had a significantly smaller PT and larger SS than other two types both pre- and post-operatively; moreover, pre-operative SA of the adjacent level was higher in the retrolisthesis group. Although the subtypes of Roussouly types among different ASD groups were varied, no significant difference of PI or ∆PI-LL was identified. It can be extrapolated from our results that patients with a more retroverted and vertical pelvis, represented by higher PT and lower SS, are bearing more loading forces at the cranial segments and are prone to develop into disc space narrowing or forward slippage of ASD after spinal fusion. Inversely, for those with more anteverted pelvis with lower PT, one of the common mechanisms in maintaining the optimal congruence between pelvic and lumbar spine is keep extension of the lower lumbar, which would impose backward shearing forces at the adjacent level and predisposed to the retrolisthesis type of ASD, especially in patients with larger local lordosis angles and more tilted endplates at the cephalad adjacent levels.

Several inherent limitations of the present study should be considered. First, the retrospective design of our study incurred certain limitations. Second, the postoperative 2-year was relatively short for the follow-up. Thus, future prospective studies with 5 to 10 years follow-up are warranted to the evaluation of ASD. Moreover, MRI was not obtained routinely during follow-up period, which would provide more standardized assessment of disc status. To be noticed, sagittal alignment was not the only factor affect the development of ASD, other multiple factors, such as BMI, facet joint orientation, muscle strength, and excessive disc space distraction also should be taken into consideration.

## Conclusions

The sagittal spinal profiles and spinopelvic parameters were significantly associated with radiological ASD, and Roussouly Type, PT, and ∆PI-LL were identified as independent risk factors; however, their influences on the development of different radiological degenerative patterns of ASD on sagittal plane were dissimilar. In those with retrolisthesis type of ASD, Roussouly Type-1 was most commonly seen, which was owing to the high stresses bear at the adjacent segment of this junctional area; while, patients with Type-2 spine and high PT were predisposed to anterolisthesis type or disc space narrowing of ASD.

## Data Availability

All data generated or analyzed during this study are included in this published article.

## Abbreviations

ASD: Adjacent segment degeneration
PLIF: Posterior lumber interbody fusion
TLIF: Transforaminal lumber interbody fusion
PI: Pelvic incidence
PT: Pelvic tilt
SS: Sacral slope
LL: Lumbar lordosis
SA: Segmental angle
MRI: Magnetic resonance image
